# Quality control, analysis and secure sharing of Luminex® immunoassay data using the open source LabKey Server platform

**DOI:** 10.1186/1471-2105-14-145

**Published:** 2013-04-30

**Authors:** Josh Eckels, Cory Nathe, Elizabeth K Nelson, Sara G Shoemaker, Elizabeth Van Nostrand, Nicole L Yates, Vicki C Ashley, Linda J Harris, Mark Bollenbeck, Youyi Fong, Georgia D Tomaras, Britt Piehler

**Affiliations:** 1 LabKey Software, Seattle, WA, USA; 2 Statistical Center for HIV/AIDS Research & Prevention (SCHARP), Fred Hutchinson Cancer Research Center, Seattle, WA, USA; 3 Duke Human Vaccine Institute, Duke University Medical Center, Durham, NC, USA; 4 Department of Medicine, Duke University Medical Center, Durham, NC, USA; 5 Department of Surgery, Duke University Medical Center, Durham, NC, USA; 6 Vaccine and Infectious Disease Division, Fred Hutchinson Cancer Research Center, Seattle, Washington, USA; 7 Department of Molecular Genetics and Microbiology, Duke University Medical Center, Durham, NC, USA; 8 Department of Immunology, Duke University Medical Center, Durham, NC, USA

## Abstract

**Background:**

Immunoassays that employ multiplexed bead arrays produce high information content per sample. Such assays are now frequently used to evaluate humoral responses in clinical trials. Integrated software is needed for the analysis, quality control, and secure sharing of the high volume of data produced by such multiplexed assays. Software that facilitates data exchange and provides flexibility to perform customized analyses (including multiple curve fits and visualizations of assay performance over time) could increase scientists’ capacity to use these immunoassays to evaluate human clinical trials.

**Results:**

The HIV Vaccine Trials Network and the Statistical Center for HIV/AIDS Research and Prevention collaborated with LabKey Software to enhance the open source LabKey Server platform to facilitate workflows for multiplexed bead assays. This system now supports the management, analysis, quality control, and secure sharing of data from multiplexed immunoassays that leverage Luminex xMAP® technology. These assays may be custom or kit-based. Newly added features enable labs to: (i) import run data from spreadsheets output by Bio-Plex Manager™ software; (ii) customize data processing, curve fits, and algorithms through scripts written in common languages, such as R; (iii) select script-defined calculation options through a graphical user interface; (iv) collect custom metadata for each titration, analyte, run and batch of runs; (v) calculate dose–response curves for titrations; (vi) interpolate unknown concentrations from curves for titrated standards; (vii) flag run data for exclusion from analysis; (viii) track quality control metrics across runs using Levey-Jennings plots; and (ix) automatically flag outliers based on expected values. Existing system features allow researchers to analyze, integrate, visualize, export and securely share their data, as well as to construct custom user interfaces and workflows.

**Conclusions:**

Unlike other tools tailored for Luminex immunoassays, LabKey Server allows labs to customize their Luminex analyses using scripting while still presenting users with a single, graphical interface for processing and analyzing data. The LabKey Server system also stands out among Luminex tools for enabling smooth, secure transfer of data, quality control information, and analyses between collaborators. LabKey Server and its Luminex features are freely available as open source software at http://www.labkey.com under the Apache 2.0 license.

## Background

Multiplexed bead arrays allow researchers to perform immunoassays that test tens or even hundreds of analytes against each sample in each plate well [1-18]. At present, many [19-23] (but not all [24,25]) of these arrays leverage Luminex® xMAP® technology, so we refer to them here as “Luminex” assays. These include both commercially available assay kits and custom assays, such as the binding antibody multiplex assay (BAMA) for human immunodeficiency virus 1 (HIV-1) developed by the Tomaras Laboratory at Duke University [5]. Such multiplexed assays can speed experimental efforts, increase lab efficiency and consume smaller amounts of sample material than ordinary enzyme-linked immunosorbent assays (ELISAs) [7,10,11,15,26]. In an ELISA, each analyte must be tested with a separate aliquot of sample in a separate well. Although Luminex assays can offer experimental advantages, they can also pose challenges in analysis [27] and quality control [15-17,28-30], particularly given the greater complexity and dimensionality of such assays than ordinary ELISAs. The continued evolution of analysis and quality control techniques, the limits of current tools and the increasingly important role these assays play in certain fields of biomedical research all make enhanced software tools desirable for management and analysis of Luminex immunoassay data.

The field of vaccine immunology provides an example of the growing importance of Luminex assays and the need for better software support. In this field, samples from vaccine trial participants can be too scarce to assay against large numbers of analytes using ordinary ELISA techniques [10,11]. To maximize the insights gained from vaccine trials, researchers have started to rely on multiplexed Luminex methods [1-3,9-14]. Recent follow-up studies for the ALVAC-AIDSVAX trial, the first vaccine trial ever to demonstrate some degree of vaccine efficacy against HIV-1 [1,31], heavily used Luminex assays. Several of these studies used Luminex assays to examine the binding of plasma immunoglobulins to panels of HIV-1 envelope proteins to determine immune correlates of vaccine efficacy [1,5].

While completing ALVAC-AIDSVAX follow-up studies, several collaborating teams found that the existing software for analysis and quality control of research Luminex immunoassays did not meet their needs in two areas. First, labs found it necessary to move data between multiple software tools to fully process all experimental data, employ advanced analytical techniques, and perform quality control across runs and reagent/bead lots. This was labor-intensive, introduced additional opportunities for error, and multiplied versions of data and analyses. Second, existing Luminex tools did not enable smooth handoff of quality-controlled data from labs to central network statisticians and principal investigators, nor provide transparency into data excluded from analysis by the labs as part of quality control.

The inherent complexity of multiplexed immunoassays makes robust, transparent quality control techniques particularly vital to achieving reproducibility, reliability and comparability of such assays [8,15-18,32-35]. Custom immunoassays that use Luminex technology, such as BAMA, can be highly reproducible; for example, BAMA has been validated for use in analyzing HIV-1 specific antibody responses in clinical trials [4,36], data in preparation for publication by Georgia Tomaras]. Nevertheless, variability in assay execution, analysis, and results is currently considered a key inhibitor of the reliable use of commercially available multiplexed assays in clinical applications, such as diagnostic biomarkers or surrogate endpoints for clinical trials [15,17,18,35,37-39]. One recent study of variability in multiplexed cytokine assay results showed significant variation across every participating lab and/or material lot for every reagent kit and multiplex platform employed (including several Luminex systems) [17]. Large-scale efforts to develop assay “harmonization” guidelines [37-42] emphasize the importance of facilitating and sharing quality techniques that can be used to overcome intra- and inter-assay variability and stabilize assay performance across labs. Growing efforts to develop reporting standards for immunoassay protocols and results [42-46] further emphasize the importance of clearly tracking and reporting variables (particularly quality control procedures) that affect assay consistency.

**Figure 1:**
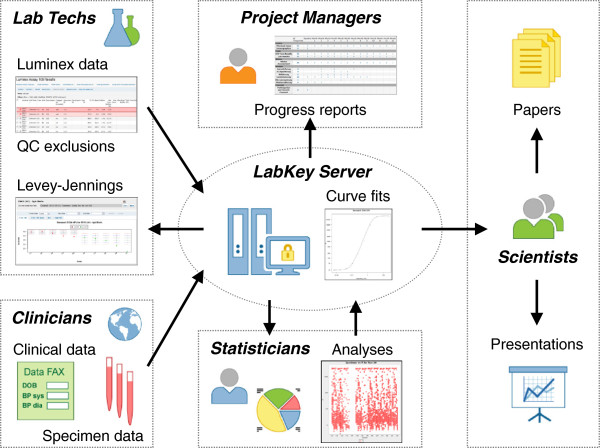
**LabKey server data flows.** This figure shows how Luminex data, quality control (QC) information, analyses and visualizations might flow between collaborating clinicians, lab technicians, statisticians, study project managers, and scientists. In this way, LabKey Server can facilitate the smooth, secure flow of information between collaborating teams during large-scale immunological studies, such as HVTN’s recent immune correlates work. Upon publication of study results, the anonymized data, analyses and visualizations produced by such a scientific team can be shared publicly by simply changing permission settings.

In response to the limitations of existing software tools, the HIV Vaccine Trials Network (HVTN) and the Statistical Center for HIV/AIDS Research & Prevention (SCHARP) at the Fred Hutchinson Cancer Research Center collaborated with LabKey Software to enhance the freely available, open source LabKey Server system [47,48] to support management, analysis and quality control of Luminex immunoassay data. Data may be produced by either customized or commercially available (kit-based) assays. The software may be particularly helpful to research organizations who seek greater control over analysis and quality control techniques; to consolidate, simplify and standardize calculation and monitoring procedures; and (as shown in Figure 1) to facilitate secure collaboration.

### Steps for a Luminex immunoassay

To provide context and terminology for software discussions, we briefly sketch an abbreviated set of experimental, analysis and quality control steps that might be used as part of an immunoassay based on Luminex xMAP technology. Note that experimental steps, materials and terminology vary with the type of immunoassay; similarly, analysis and quality control techniques continue to be active areas of research [15,16,27-30,32,34].

**Figure 2:**
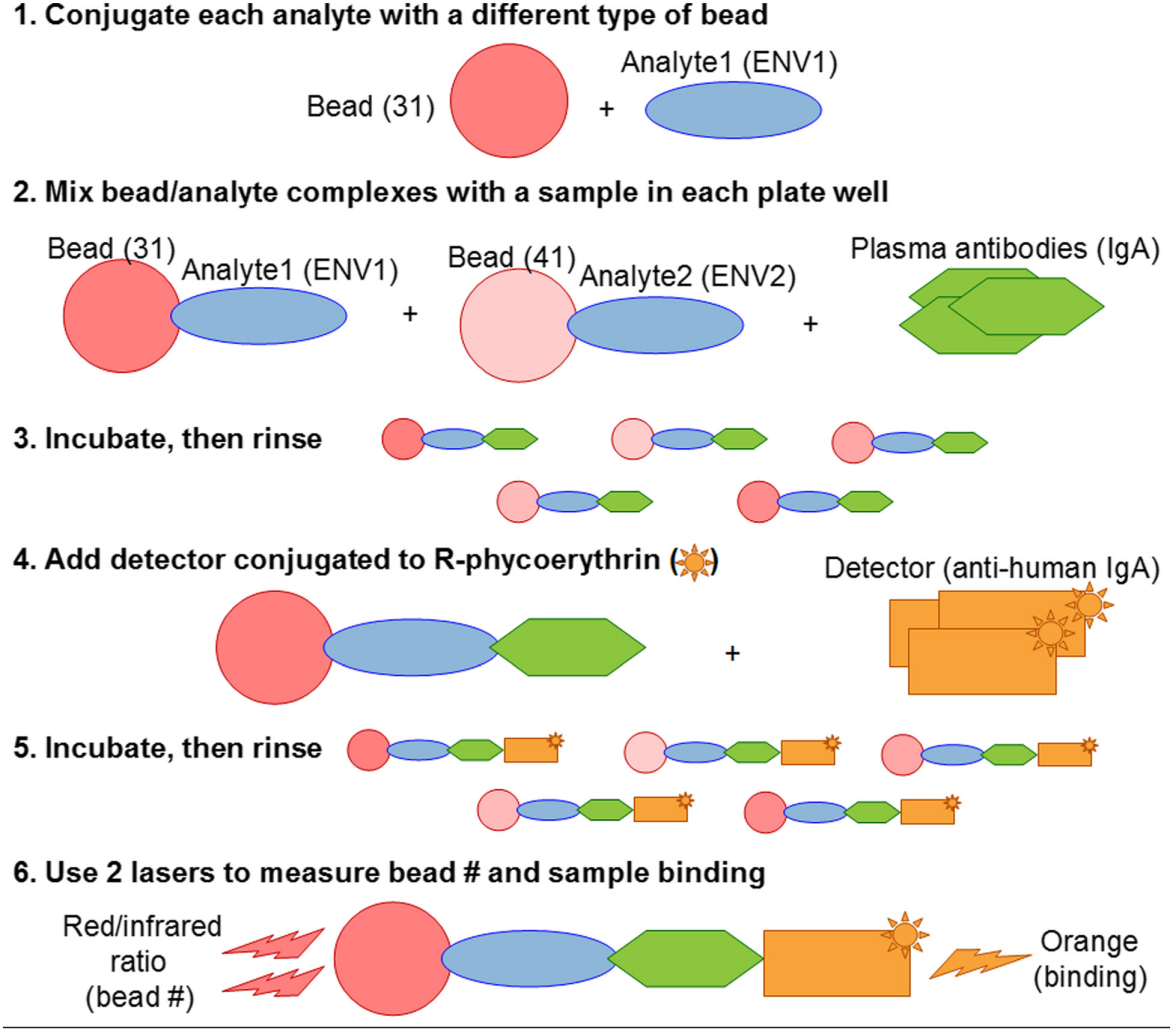
**Luminex immunoassay.** This figure sketches an experimental process that might be used in a Luminex immunoassay used to evaluate the binding of a panel of HIV envelop proteins to plasma IgA antibodies. The figure does not include analysis or quality control steps.

Figure 2 shows how you might evaluate the binding of plasma antibodies from study participants (e.g., immunoglobulin A (IgA) antibodies) to a panel of analytes (e.g., virus envelope (ENV) proteins (antigens)). Experimental steps:

(1) ** Conjugate each analyte with a different type of bead.** Each bead type contains a mixture of red and infrared dyes whose ratio can produce a spectral signature that uniquely identifies the bead type (and thus the analyte).

(2) ** Mix bead/analyte complexes with a sample in each plate well.** Sufficient numbers of each type of bead must be deposited in each well to satisfy thresholds for statistically significant results. Optionally, unbound (“blank”) beads may be included in the bead mixture to allow subtraction of fluorescence from non-specific binding to beads. The type of sample deposited in each well varies depending on the purpose of the well: (i) A titrated *standard*, whose concentration is known and used to calculate a reference curve (ii) A titrated *quality control*, whose concentration is known and used to calculate a reference curve (iii) An *unknown*, such as serum from a study participant (iv) A positive or negative *control* (v) A *background* well, which contains only buffer and is used for subtracting that results from non-specific binding of the detector. Samples are usually replicated in several wells.

(3) ** Incubate, then rinse.** This removes excess sample.

(4) ** Add detector conjugated to R-phycoerythrin.** The detector and its attached fluorochrome later serve as reporter for the binding of sample material to the bead/analyte complex. The detector is sometimes called the *conjugate* for the experiment.

(5) ** Incubate, then rinse.** This removes excess detector.

(6) ** Use two lasers to measure bead number and sample binding.** Typically, flow cytometry and two lasers are used to make single-bead measurements. For each bead, one laser excites the bead’s dye, allowing identification of the bead type (and thus the analyte) from the red/infrared ratio of light, as well as detection of beads of incorrect size (such as doublets or fractured beads). A second laser excites the detector-bound fluorochrome, allowing measurement of sample binding. For each type of analyte in each well, the instrument reports the median fluorescence intensity (FI) for the bound detector for all beads coupled with that analyte.

On a per-run basis, analysis and quality control of results may include: normalization to account for background fluorescence, exclusion of suspect data, calculation of dose–response curves for titrated standards, the use of these curves to interpolate estimated concentrations of unknowns, and other steps. Performing quality control across many assay runs may involve: calculating representative metrics for each standard curve for each run, plotting these metrics and expected ranges on Levey-Jennings [49] charts to determine outliers, excluding data from analysis based on quality metrics, and identifying trends that require further investigation.

## Implementation

### Architecture

LabKey Server is a web application implemented in Java. It runs on the Apache Tomcat web server and stores its data in a relational database, either PostgreSQL or Microsoft SQL Server. LabKey Server has been tested on computers running Microsoft Windows and most Unix variants, including Linux, Macintosh OSX and Solaris.

LabKey Server’s tool for Luminex is packaged in a Java-based module that encapsulates user interface elements and calculation logic for designing, processing and displaying structured assays of various kinds. The tool can leverage a customizable transform script that performs Luminex-specific analysis calculations during import of assay data. If included, this script is also re-run automatically when data from a titration are excluded from analysis because the exclusion affects the analysis results. The default Luminex transform script is written in R, but many other programming languages could be used (e.g., Perl, Python, or Java).

Details of LabKey Server’s architecture and assay module are covered elsewhere [47]. LabKey Server v12.3, available in December 2012, is the 25^th^ official, public release of the platform since 2005.

### Configuration of scripting

To access all features of the LabKey Server tool for Luminex, the R scripting environment must be installed and configured on the server. To leverage the default Luminex transform script (*labkey*_*luminex*_*transform*_*script*.*R*), one must also install the script, its utility script (*youtil*.*R*), the Ruminex R package [27], and required R packages (Rlabkey, xtable, drc and Cairo). See Additional file 1 to obtain the first three; the other R packages are available from the Comprehensive R Archive Network (CRAN) [50].

## Results

### Overview of the Lumimex tool

When a researcher imports Luminex run data into a LabKey Server, the researcher is prompted to enter relevant metadata about the batch of runs, the run itself, included analytes and relevant titrations. Metadata include information required for calculations (e.g., normalization options, and desired standard for each analyte) and experimental characteristics that can be used for tracking purposes (e.g., experiment performer and reagent lot number). The system can be configured to use an R script to automatically analyze raw data and metadata to determine dose–response curves for titrations, estimate concentrations using these curves, identify outliers, determine quality control metrics for curves and plot curves. Unreliable experimental data can be flagged for exclusion from analyses. When a transform script is configured, the system automatically provides Levey-Jennings quality-control charts and allows users to define expected ranges using baseline sets of runs. Assay results can be integrated with other data types, such as specimen information and clinical data for study participants. The Luminex tool displays all source data, quality control exclusions, calculated values and curves in a secure, interactive, web-based interface.

### Options for leveraging the tool

LabKey Server’s tool for Luminex can be accessed in two ways:

• By installing and configuring a LabKey Server instance. This option allows you to administer and customize a private LabKey Server for your lab or organization. The “Availability and Requirements” section of this paper explains how to obtain installers and/or source code.

• By logging into to the Atlas Science Portal [51]. This option is available to qualified researchers at many of the consortia participating in the Global HIV Enterprise [47,52,53]. To inquire about access, contact atlas@scharp.org.

### Documentation, tutorials, live demo, and context-sensitive help

Full documentation and tutorials for setting up, configuring and using LabKey Server and its Luminex assay tool are available at http://www.labkey.org. This documentation is updated regularly to match the currently released version of LabKey Server. The basic and advanced Luminex tutorials [54,55] provide detailed walk-throughs of Luminex setup and scenarios. They also include a troubleshooting guide [56] that helps users interpret and fix error messages from Luminex transform scripts, plus address problems with script-calculated values, such as curve fits. The tutorials are accompanied by live demos [57] that allow visitors to interact with Luminex features that do not require editor-level permissions. For each page in the Luminex workflow, the software provides a context-sensitive **Help** link in the upper right corner that leads to a relevant tutorial or documentation page.

### Assay file formats

LabKey Server understands Microsoft Excel® files output by Bio-Plex Manager™ [58] for each Luminex assay plate run. Bio-Plex Manager is software that runs several types of Luminex devices. The LabKey Server tool for Luminex has been tested with the results of 96-well plate assays. It is expected to be compatible with newer, larger plates, such as those with 384 wells.

Each run file includes a separate worksheet for each type of bead (and thus each analyte). Each sheet displays a header with run metadata, one or two data tables, and a footer with calculated parameters and flags. Data tables report results provided by the instrument, either on a per-well basis (raw data) or on a per-sample basis (summary data). If summary data are reported, each row supplies an average for all sample replicates. Some files contain both raw and summary data tables.

Each row in a data table reports the sample type (background, standard, control or unknown), sample identifier, well location (for summary data, multiple are shown), sample description (often an identifier that can map the sample to its source specimen and study participant), FI (fluorescence intensity averaged across replicates for summary data), FI minus mean background well FI, coefficient of variance, expected concentration (for titrations only), dilution, bead count (for raw data only), and other columns.

### Usage scenario for the tool

**Figure 3:**
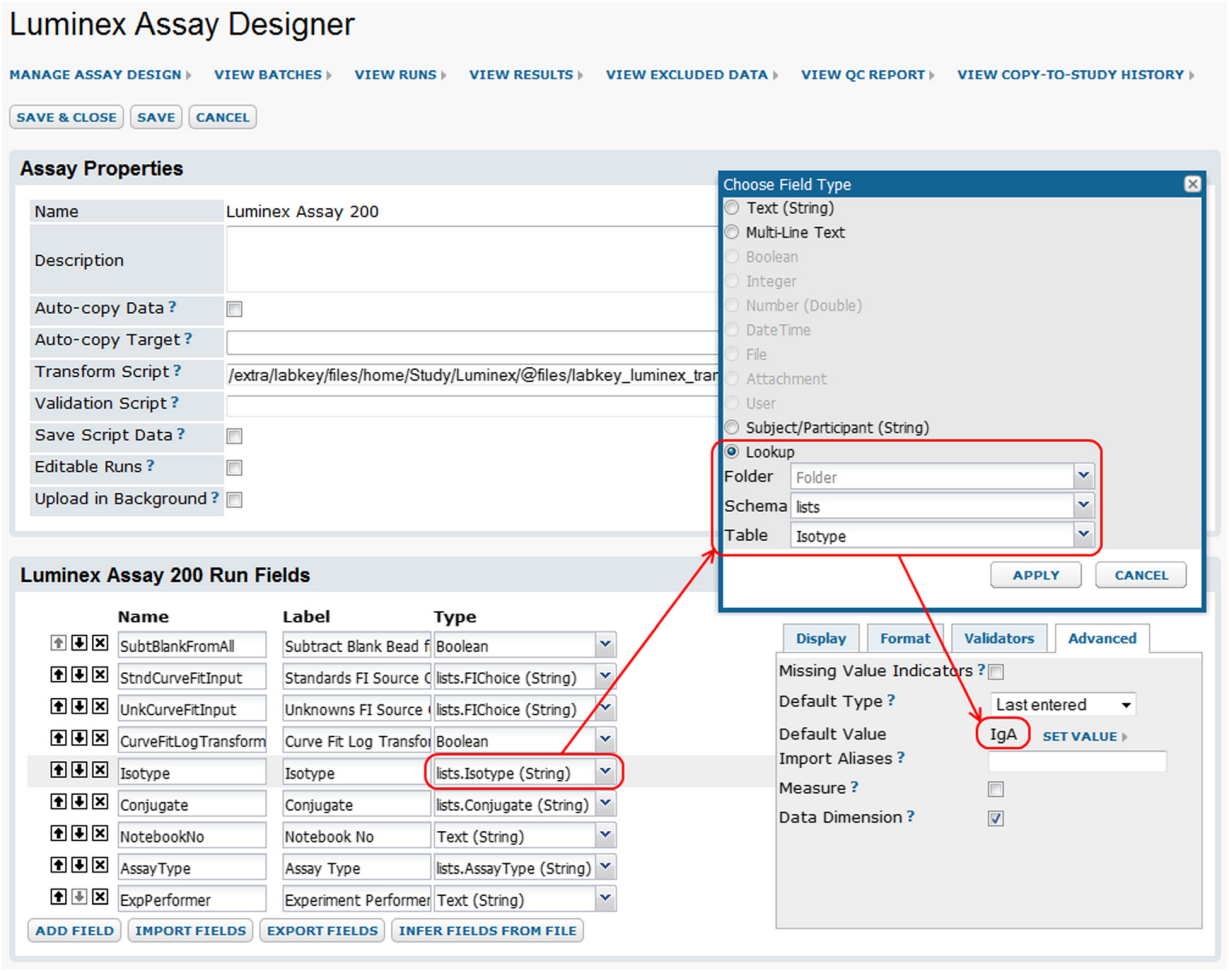
**Assay design editor.** An assay design describes the data fields imported from a run data file, the metadata collected from the user during import, and other properties of the assay, such as the location of the transform script run during data import. For each field, the design can define an expected range; default value; acceptable values; conditional formatting; display formatting; editability; and a range of other settings [47]. Custom, lab-specific fields can be defined at the batch, run, and analyte levels. If the transform script is customized to output new fields (such as calculated values) and these fields need to be stored in the database, corresponding destination fields must be added to the assay design. This screenshot shows a portion of an assay design and illustrates how customization of such a design can facilitate collection of run-specific data from the user during data import. Here, the **Isotype** run metadata field (highlighted in gray) has been defined as a lookup to a pre-defined list (a simple table). This list, also called **Isotype**, populates a drop-down list of data entry options that are presented to the user during run import (as shown in Figure 4). The **Isotype** field has been further customized to default initially to *IgA* (one of the options defined by the lookup list), then after that to the *Last entered* value.

**Figure 4:**
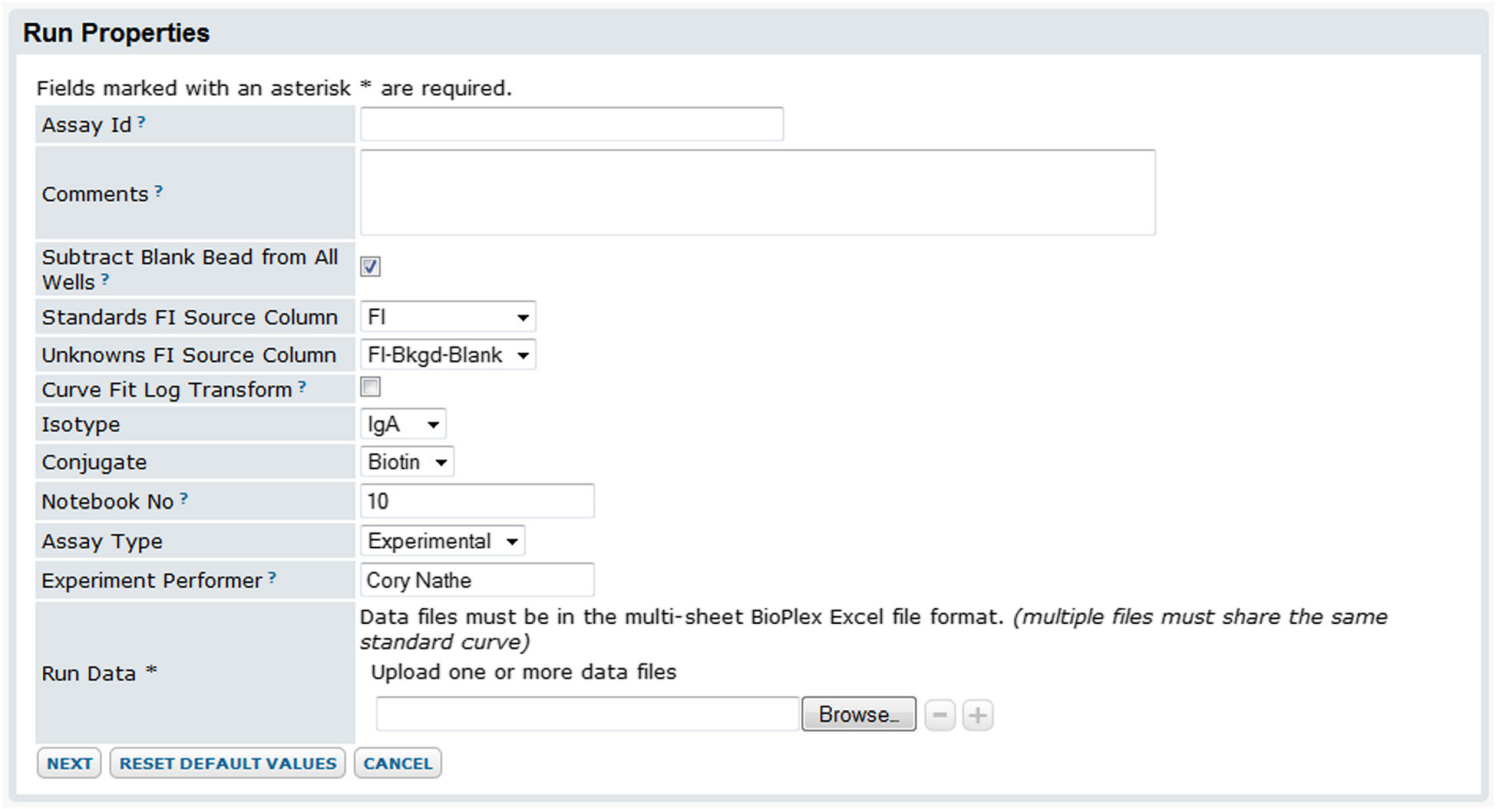
**Run properties data entry form.** This image shows an example of a data entry page for run metadata provided to the user as part of the run import process. It illustrates how pre-set defaults and lookups defined in an assay design (such as the one shown in Figure 3) can ease and standardize data entry during run import. Here, the **Isotype** field shows a default of *IgA* and provides a drop-down list of other options. Both the default and list of options were configured in the assay design shown in Figure 4. The use of a standardized vocabulary for data entry makes it easier and more accurate to aggregate, filter and report on the information entered. The use of default values can speed data entry when the same values apply to many runs.

**Figure 5:**
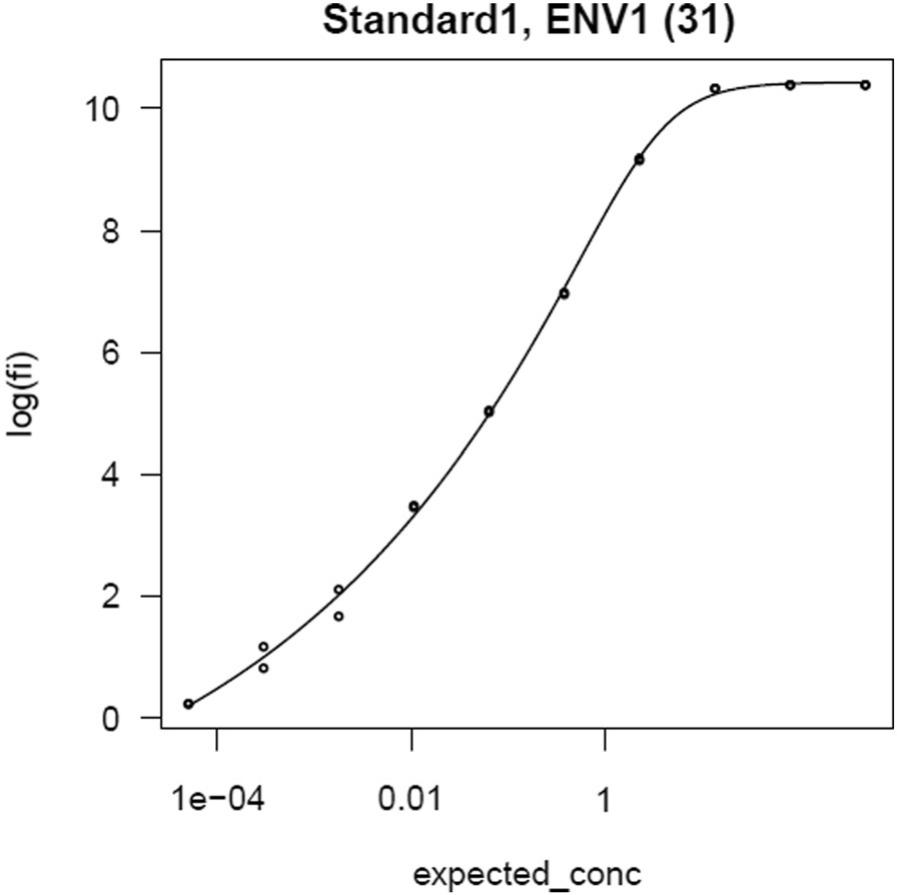
**Standard titration curve visualization.** The system provides a PDF file that contains a plot for each curve fit calculated by the transform script for each standard or quality control titration. The plot also displays the source data used to calculate the curve.

**Figure 6:**
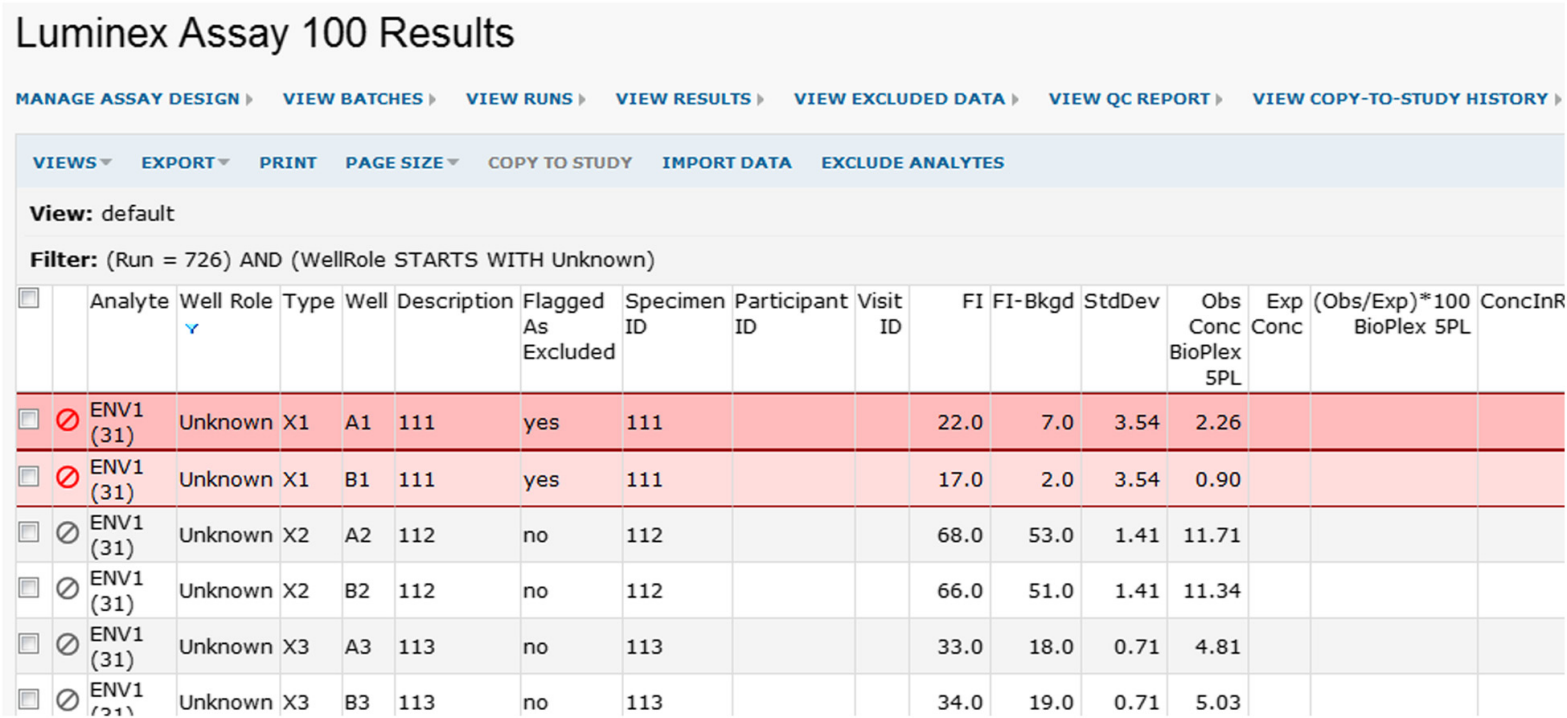
**Highlighting for excluded wells.** When a user excludes data from analysis (either on a per-replicate-group or per-analyte basis), the excluded rows of data are flagged with red highlighting in the user interface, as shown here.

**Figure 7:**
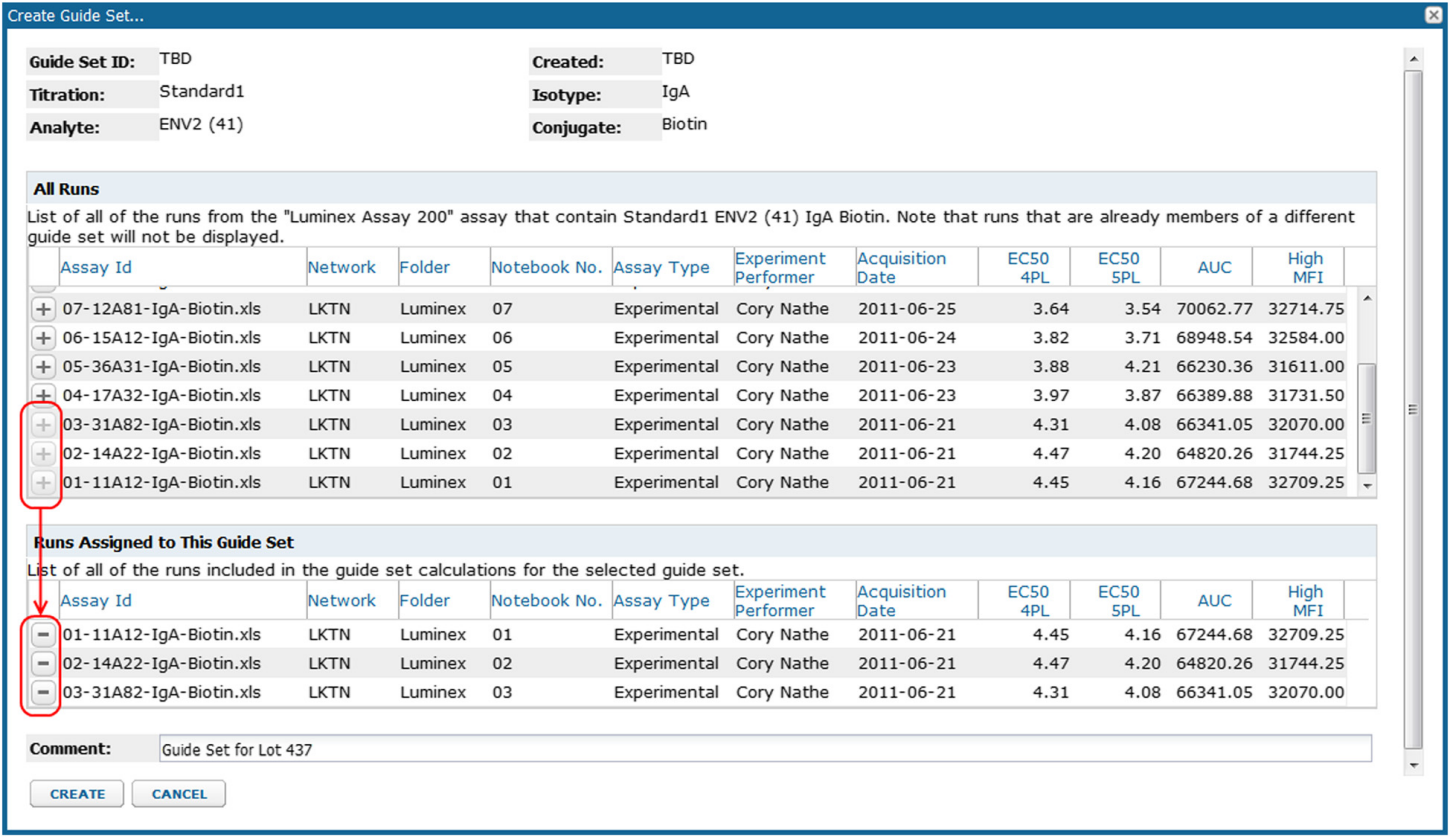
**Creation of a guide set.** This figure shows how baseline runs are incorporated into a “guide set” used to define expected value ranges for analyte standards and quality controls for runs that share a common lot of reagents (e.g., beads).

**Figure 8:**
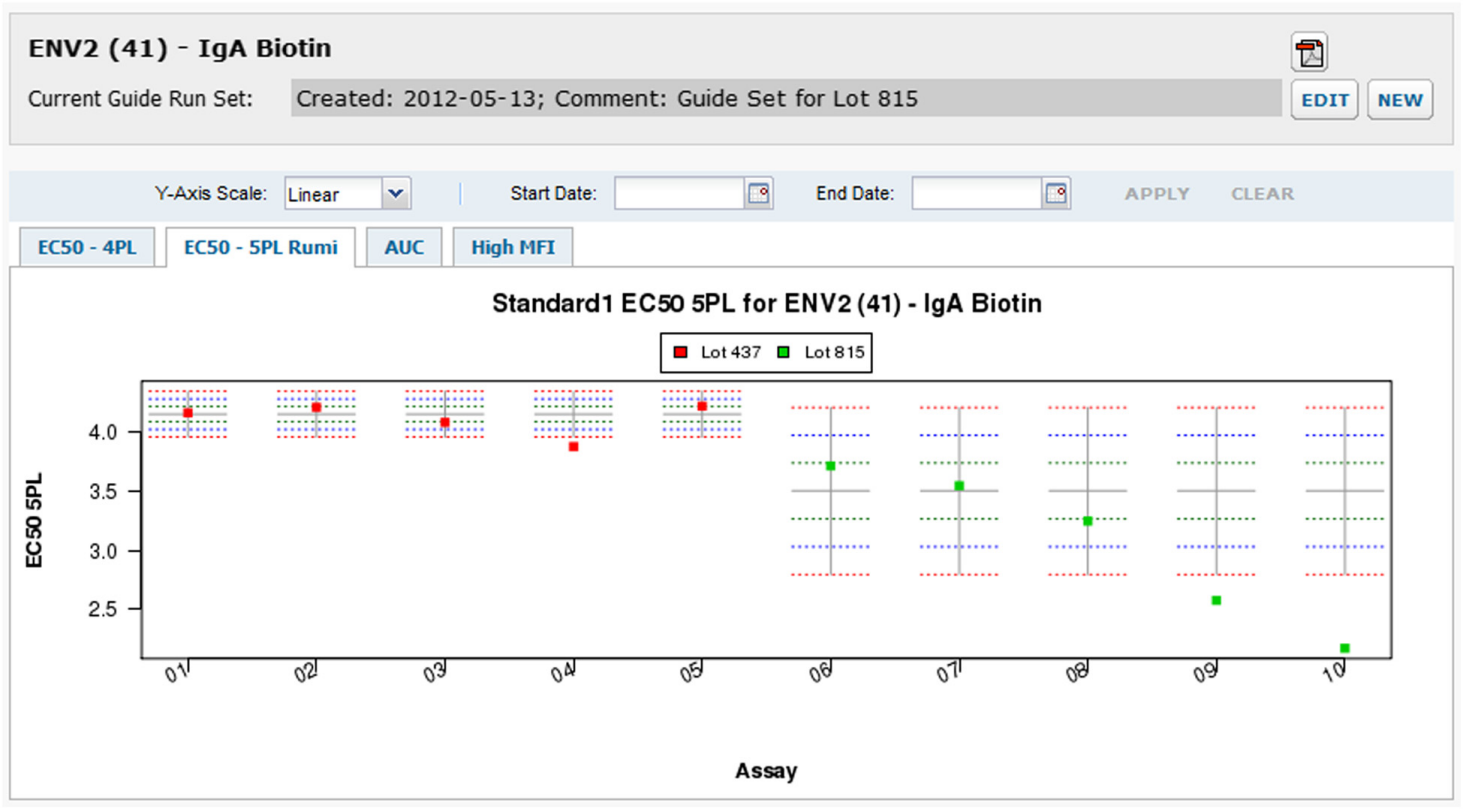
**Levey-Jennings chart for cross-run quality control.** Levey-Jennings charts can help visualize changes in a quality control metric over time, across runs, and across material lots. This Levey-Jennings chart shows expected ranges for EC50s for standards run for the ENV2 analyte. The transform script calculated these EC50s using the Ruminex package and 5pl curve fits, so they are displayed on the EC50 – 5pl Rumi tab of this interface. This example shows differences in expected EC50 ranges for two groups of runs, each of which is associated with a different lot of experimental materials. The example also shows a trend over time in EC50, which might suggest decay in experimental conditions or materials. Such decay would require further investigation. It might require exclusion of results from certain days or material lots, replacement of old reagents, investigation of material supply consistency, or other such measures. Early identification of such a trend can allow a lab to take corrective measures before a quality metric falls outside of acceptable bounds and assay runs must be rejected. Outliers visible in this plot are flagged for review, as shown in Figure 9. Charts can be exported to PDF using the PDF icon.

**Figure 9:**
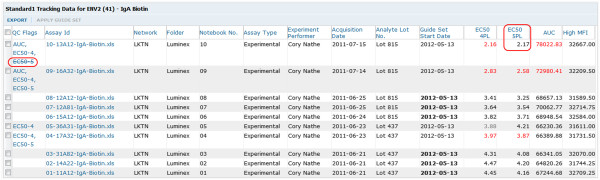
**Active and inactivated quality control flags.** Metrics used for cross-run quality control (4pl and 5pl EC50s, AUC and HighMFI for standard or quality control titrations) are flagged for review when they fall outside the maximum expected range. As visualized in Figure 8, the maximum expected range around the mean for a metric is +/−3 times the standard of deviation of the relevant “guide set” (benchmark group of runs). Flagged metrics are highlighted in red, plus listed in a separate column, which facilitates review. Users can inactivate quality control flags for metrics that have been reviewed and deemed acceptable. Once a metric for a run has been inactivated, its red highlighting is removed and its listing is crossed out, as shown here for the 5pl EC50 for the run at the top of the list.

**Figure 10:**
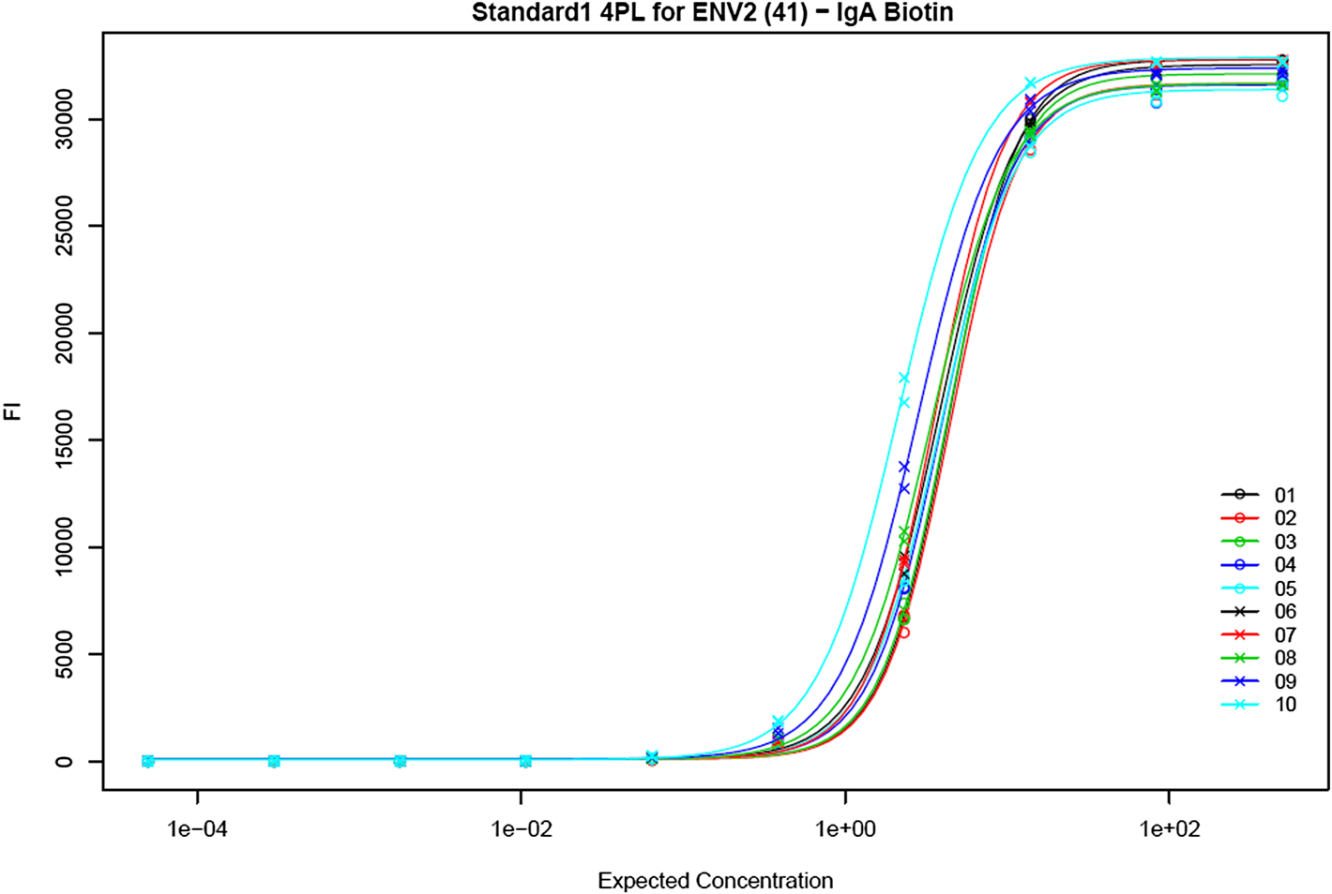
**Multi-curve plots of standard titrations for several runs.** Plotting standard curves for several runs together (*a*.*k*.*a*., creating a curve “graveyard” [15]) helps visualize any inconsistencies in data and curve fits between runs. The curves shown here are 4pl standard titration curves for the same data used in Figures 7, 8 and 9. Users can select which curves to plot together. Plots are provided for both FI and log(FI), but only FI is shown here.

To analyze and perform quality control on Luminex data, a lab might follow these steps:

1. **Set up an assay project.** After setting up a LabKey Server, an administrator creates an assay-type project on the server with appropriate permissions for user access. It provides a central location for managing descriptions of Luminex assays and associated run data.

2. **Create an assay design.** An assay design [47] both describes the content expected for a particular type of assay run file and provides a framework for importing many runs and their associated metadata in a standardized manner. An administrator can either select the default Luminex assay design, or import a Luminex assay design archive that is tailored to support the default transform script. Both designs can be further customized, as shown in Figure 3. When usage of a transform script is desired, the path to this script is entered in the assay design.

3. **Import data and enter metadata.** After an administrator has created an assay design in the assay project, users can import run data to the server. As part of this process, the assay design guides the collection of appropriate metadata for the assay run, as shown in Figure 4. Certain metadata are used to control Luminex-specific data processing. For example, information collected may determine processing steps for inputs to calculations (such as normalizations for background fluorescence), identify the standard(s) used for calculating interpolated concentrations, or associate analytes with metadata (such as bead lots). Other metadata are used to annotate the run, run analytes, and/or batch of runs. During import, the transform script automatically performs calculations on the data and the system flags certain data for quality control (as discussed below in the “Calculations” section). Several files may be imported together as part of one run, allowing standards in one file to be associated with results in another.

4. **Explore results.** Curve fits and related results are calculated automatically by the transform script for each run during the import process. Results include curve fit parameters, estimated concentrations interpolated from calculated curve fits for standard titrations, and metrics calculated from curve fits, including EC50 (effective concentration at 50% of the difference between the asymptotes of a dose–response curve), AUC (trapezoidal area under the curve) and HighMFI (highest mean FI). Calculated results and data from run files are displayed together in grid views. The system provides curve visualizations based on both 4- and 5-parameter logistic regressions (4pl and 5pl), as shown in Figure 5. Users can explore their data through sorting, filtering and customizing grid views; using built-in visualization and analysis tools; or building custom SQL queries or visualizations [47]. For example, LabKey Server’s built-in R scripting environment allows statisticians to define R scripts that produce visualizations and associate them with certain kinds of results.

5. **Perform within-run quality control.** Questionable results can be excluded from analysis on a per-analyte or per-replicate-group basis. If a replicate group of wells for a titration is excluded, the transform script is re-run to recalculate curves and associated results. Figure 6 shows how the system applies red highlighting to all excluded data.

6. **Perform cross-run quality control.** The system automatically plots Levey-Jennings charts for four metrics of performance (4pl EC50, 5pl EC50, AUC and HighMFI) for both standards and quality controls chosen for each analyte. Users can select a suite of baseline runs (a “guide set”) to establish expected ranges for analyte-specific standard metrics for all runs associated with the same lot of experimental materials, as shown in Figure 7. These ranges (+/− one, two and three standard deviations) are displayed on the Levey-Jennings plot for each metric for the analyte standard, as shown in Figure 8. For each run associated with the same lot of materials, quality control metrics that fall outside of expected ranges are flagged for review, as shown in Figure 9. Standard curves for selected runs can be displayed together to help identify inconsistencies in curves across runs, as shown in Figure 10.

7. **Share quality-controlled data with collaborators.** After a lab has completed quality control for a set of runs, a lab data manager copies finalized data into a shared, study-type project. A study-type project serves as an integration and analysis hub for many types of related data for a study (e.g., specimen information, study participant demographics and results from other assays) [47,59]. Collaborators with appropriate permissions can use the server’s web portal to view and analyze this data. Administrators can set up custom views or SQL queries that allow different parties (such as lab technicians, statisticians and project managers) to see the data in their own preferred formats. Role-based, inheritable and customizable permission settings [47] support controlled, secure sharing within a lab, between labs, between organizations, and/or with the general public. Collaborating scientists can use the shared data portal to bring different perspectives to results, explore alternative analysis approaches, and collaborate efficiently on publication.

8. **Publicly share data and analyses as part of a publication.** Upon publication of results in a scientific journal, specified subsets of anonymized, releasable data can be publicly shared on a LabKey Server web portal alongside contextual information and tools, including quality control information and analytical scripts. Providing public, interactive access to publication-related data allows a wider pool of scientists to explore the results and investigate alternative analytical approaches. Interactive release of data, quality control information, and analytical tools also supports study reproducibility [35].

### Calculations

Calculations performed on Luminex data fall into three categories: automatic calculations performed by the server, automatic calculations performed by the transform script, and optional calculations performed by the script. Calculations by the script only occur if the script is associated with the assay design. Calculations by the system and the script are performed during data import. Additionally, if data for a replicate group of wells in a titration are excluded, the transform script and its calculations are re-run.

**Automatic calculations and flagging performed by the server.** LabKey Server provides automatic calculation of coefficients of variation (CVs) and provides flagging for outliers of various kinds to facilitate human review.

For quality control of multiplexed immunoassays, researchers typically pre-select acceptable thresholds of variation across replicates and use these thresholds to flag, review, and exclude dubious data in a standardized fashion. CVs are used to measure variability for different kinds of replicates (samples, assay runs, material lots, analysis labs, etc.) and identify questionable outliers or patterns that may be artifacts of experimental conditions [15,17,33,34,60]. Outliers can bias top asymptotes, bottom asymptotes, and slope parameters [60,61]. CVs for both intra- and inter-assay measures may also be monitored over time to identify changes in experimental conditions that may affect the accuracy and comparability of results [15].

When the imported run file does not include summary data, LabKey Server calculates the CV of the FIs of each replicate group (sample/dilution/antigen combination). CVs are stored with each well-level row and measure variability across replicates. They are displayed as percents (%CVs).

LabKey Server adds a quality control flag to each data row whose reported FI is greater than 100 *and* whose %CV is greater than 15 (for unknowns) or 20 (for standards and quality controls). FI > 100 is used as a threshold to save review time; for our collaborators, only samples that are potentially “positive” (significantly changed with respect to a baseline) warrant further investigation. Columns flagged through this process are highlighted in red.

LabKey Server can automatically provide additional flagging and reporting of outliers. Assay design fields can be configured with arbitrary conditional formatting settings that automatically color code values when viewed in the user interface or in Excel file exports. A user can set up saved, custom views that automatically filter results to show only data rows that exceed user-set thresholds for user-selected measurements. Furthermore, as mentioned above, the system’s analyte- and replicate-specific exclusion options can be used to systematically add custom flags and exclusions, which the server displays in summary reports.

**Automatic calculations performed by the default transform script.** The script automatically applies a conversion function to normalized FIs to account for negative values potentially introduced by the removal of blank bead and/or background fluorescence. Currently, the script calculates: converted FI = max(FI, 0) + 1.

When calculating 5pl curve fits, the default transform script uses the Ruminex R package [27], which in turn uses the drc R package [62]. When calculating 4pl curve fits, the script uses the drc R package directly. Users can edit the transform script to use alternative strategies for performing 5pl and 4pl curve fits [27,63,64], including using custom seed and optimization functions. Users can also add new calculations to the transform script.

**Optional calculations performed by the default transform script.** The default transform script enables users to opt to:

• Normalize FI by subtracting blank bead and/or background well FI before calculating curves or interpolating unknowns. This subtracts fluorescence signal that is due to non-specific binding to the beads.

• Log transform normalized FIs before calculation of curve fits, which can help stabilize variance extremes [27].

• Apply weights to the squared errors used in goodness of fit optimization calculations. Weighting can help account for lower variance at lower FI (heteroscedasticity). This improves curve fits, particularly at the low range, thus expanding the useful range of data. Using the transform script, these weights can be arbitrary (e.g., weight = 1/FI^1.8^)

• Calculate "positivity" by determining whether the value for each analyte has increased relative to a baseline value and also exceeds an analyte-specific threshold. This is helpful, for example, when you wish to determine whether measurements for an individual in a trial have increased relative to baseline, pre-trial values. Both the baseline and threshold for each analyte are entered during run import.

### Additional system features and supported technologies

Additional features of LabKey Server are likely to be particularly helpful to users of the Luminex tool. These include: a fine-grained security model; auditing; options for exporting data in a variety of formats; file upload and sharing; and client APIs (application programming interfaces) for building custom interfaces and workflows using a variety of common languages (e.g., JavaScript, Java, Python, R, etc.) [47,48,59].

LabKey Server also allows researchers to integrate assay results (such as Luminex results) with related study data [47,59]. Specimen identifiers in run data files can be mapped automatically to study-specific specimen identifiers (or participant/visit identifiers). This allows linkage and integration of specimen, experimental and clinical data.

The system also supports assays and techniques commonly used by immunologists, including ELISAs, neutralizing antibody assays [59], flow cytometry [65,66], proteomics [48,67], and next-generation sequencing [68,69]. Furthermore, LabKey Server also supports the description, management and analysis of novel assay types [47].

### Current usage

The full set of features described here only became available in April 2012 with the release of LabKey Server 12.1, so adoption of the improved Luminex assay tool has been promising. As of January 2013, the Atlas Science Portal maintained by SCHARP, HVTN and their collaborators contained a total of 1,081 Luminex runs contributed by at least three separate labs.

## Discussion

### Overview

Among software for Luminex analysis and quality control, LabKey Server stands out most distinctively in its support for customization, traceable quality control, and secure data sharing. These capabilities can help labs develop and adopt the new methods; simplify and standardize workflows; and securely, smoothly share information with collaborators. LabKey Server is also notably unique among Luminex-specific tools for being both open source and freely available (Apache 2.0 license [70]). Current, notable limitations include a lack of support for running Luminex instruments and a requirement that input run files conform to the Excel file format output by Bio-Plex instrument software.

### Alternatives

Beyond LabKey Server, only a few software tools provide Luminex-specific support, and all of those tools provide only limited customizability. A much wider range of software tools provide significant customizability alongside general-purpose statistical features, but those tools do not inherently support Luminex-specific workflows. Table 1 compares the capabilities of LabKey Server with six representative tools: three systems tailored for Luminex (Luminex xPonent™ [71], Bio-Plex Manager [58], and MiraiBio MasterPlex QT™ [72]) and three general-purpose statistical tools (GraphPad/Prism® [73], Microsoft Excel [74], and R [75]). To highlight tradeoffs, features are roughly ordered from greatest Luminex specificity to greatest scope for customization.

**Table 1:** Comparison of software tools

	***Software***						
***Features***	**Luminex xPONENT**	**MiraBio MasterPlex QT**	**Bio-Plex Manager**	**GraphPad/Prism**	**Excel**	**R**	**LabKey Server**
Graphical user interface	+	+	+	+	+	-	+
Runs a Luminex-type instrument	+	-	+	-	-	-	-
Built-in, automatic quality control flagging of some kind	+	+	+	-	-	-	+
Built-in, automatic curve fits for titrated controls and unknowns, not just standards	+	+	-	-	-	-	+
Built-in mechanism for choosing a standard from a different plate	+	+	+	-	-	-	+
Option of weighting squared errors by to 1/FI^2^	+	+	+	+	+	+	+
Can subtract blank bead fluorescence	-	-	-	+	+	+	+
Can generate Levey-Jennings plots for cross-run quality control	-	-	-	+	+	+	+
Support for scripting in some language	-	-	-	+	+	+	+
Client APIs for development of custom interfaces	-	-	-	-	+	+	+
Can weight squared errors with custom functions	-	-	-	-	-	+	+
Fully customizable 5pl algorithm	-	-	-	-	-	+	+
Open source	-	-	-	-	-	+	+
Freely available without time restrictions	-	-	-	-	-	+	+
Portal for secure data sharing	-	-	-	-	-	-	+

This table compares LabKey Server with a representative sample of software used for analysis and quality control of research Luminex immunoassays. This list aims to show how LabKey Server provides advantages over other existing software tools, not to compare all capabilities relevant to Luminex analysis. Documentation for many tools was incomplete, so the table provides only a reasonable inference of feature availability.

This table does not aim to address tools for scientific data sharing [47]. Furthermore, the table only includes Luminex-specific software designed for research assays, not software designed to support standardized diagnostic tests. Research assays typically require greater scope for cutting-edge analysis than manufacturer-defined diagnostic tests.

### Advantages

LabKey Server’s tool for management and analysis of Luminex immunoassay results can help labs and research organizations to:

(i) **Efficiently perform complex analyses of high-content data.** The nature of multiplexed immunoassays results in a significant amount of information per experimental run. The tool can help teams efficiently manage and quickly analyze the complex, multidimensional data produced by such assays.

(ii) **Facilitate networked research through smooth, secure sharing of Luminex results.** A central, shared data portal/repository can prove particularly useful to research networks and consortia who employ central data management and statistics groups to execute study-wide and/or cross-study analyses based on data pooled from collaborating labs [47,51,76]. As shown in Figure 1, labs can use such a portal to supply statisticians with data and quality control information in a secure fashion. Central data managers can use the system’s reporting tools to efficiently monitor and troubleshoot study progress. Principal investigators (and others with appropriate permissions) can gain transparency into study progress, analyses and data, as well as explore alternative analysis approaches and collaborate on publications. Using a central repository can also help prevent loss of valuable data from individual computers. After study publication, a subset of releasable, anonymized study results can be made publicly available on the organization’s data portal by changing permission settings.

(iii) **Retain raw Luminex data, quality controlled data, and analyses to make them available for later re-analysis.** LabKey Server stores raw run data from the instrument, quality control information, calculated values, final datasets, visualizations, and the original run data files. Data records that have been flagged for quality control can be excluded from analysis without deletion. When data is stored in a shared repository, statisticians, labs and principal investigators can examine or export copies of the data as it looked at different stages of processing (e.g., with and without quality control exclusions).

(iv) **Consolidate, simplify and standardize lab workflows by using one primary software tool for Luminex analyses.** Eliminating the labor-intensive steps necessary to move data between tools for different calculations can increase efficiency, foster standardization and reduce opportunities for error. If the software is deployed on a shared portal, users have access to the same version of the tool and the same analysis algorithms without the need to install updates on a multiplicity of machines [59].

(v) **Customize calculations and visualizations.** Labs can adopt new analysis and quality control techniques without adopting additional software tools or complicating workflows. They can use scripting and custom assay designs to incorporate the latest algorithms (e.g., new techniques for curve fitting), visualizations (e.g., custom R plots) and processing steps (e.g., subtraction of blank bead fluorescence) while still providing users with a friendly, graphical user interface. Labs can test out new analytical techniques and transform scripts without disrupting mainline workflows by establishing separate assay designs for exploratory analytics.

(vi) **Track quality control across runs and reagent/bead lots using Levey-Jennings plots and outlier flagging.** Both LabKey Server and existing Luminex-specific software packages enable both automatic and manual identification of outliers within runs. However, only LabKey Server provides extensive support for quality control tracking *across* runs and material lots using Levey-Jennings plots.

(vii) **Gain flexibility in plate layouts and titration analyses.** LabKey Server allows a user to associate multiple standards with each analyte, automatically perform curve fitting for all titrations (including quality controls and unknowns, not just standards), and select a standard on a different plate for use in interpolation of concentrations of unknowns. Bio-Plex Manager does not currently allow any of these options without rerunning analyses. Other software supports only some of these options.

(viii) **Leverage LabKey Server platform features.** The base system provides extensive support for data analysis, integration, visualization, reporting and workflow interface development. It also supports other assays and techniques commonly used by immunologists.

(ix) **Use open-source, freely available tools.** At present, LabKey Server is the only open source tool with a graphical user interface that provides substantial support for Luminex-specific scenarios. The R scripting environment is also open source, as are a wide variety of R statistical analysis packages. Using open source tools can provide advantages in transparency, cost and extensibility.

(x) **Facilitate assay harmonization across organizations.** Overcoming variability and reproducibility issues for commercially available, multiplexed immunoassays for cytokine measurement is considered key to broadening the viable applications of such assays [37,38]. To harmonize assay results, researchers identify experimental and analytical variables that strongly influence assay performance and use them to develop quality control guidelines that improve assay reproducibility across labs, material lots, organizations, etc. Identification of such performance variables requires sharing not just final results, but also protocol records, quality control information, data processing methodologies, and (possibly) analytical tools across collaborating organizations. To our knowledge, LabKey Server has not yet been employed for assay harmonization at a scale worthy of note; nevertheless, the system seems particularly well-suited given its support for sharing just this kind of information and tools. An installation of LabKey Server (the Atlas Science Portal [51]) has already been used for wide-scale proficiency testing (comparison of the performance of a standardized protocol across many labs) of a neutralizing antibody assay [59].

### Limitations

Current limitations on the use of LabKey Server for analysis of Luminex experiments include:

(i) **No support for running a Luminex instrument or designing Luminex plate layouts.** Software such as Luminex xPonent, Bio-Plex Manager or MILLIPLEX Analyst™ [22] is currently required for running instruments that leverage Luminex xMAP technology.

(ii) **Limited Luminex data file format compatibility.** Currently, the LabKey Server tool for Luminex only understands data files in the Excel format output by the Bio-Plex Manager software. However, the system can still infer the structure (column names and data types) of other tables of data, which allows users to quickly import other kinds of tabular data files, such as metadata files. Custom SQL queries can join imported tables of metadata to other kinds of Luminex data for display as custom data grids [47].

(iii) **Familiarity with R recommended for extensive customization of analyses.** Customization of certain aspects of data processing and analysis (e.g., selection of background FI subtraction settings) can be made through the user interface. However, making extensive changes to the default transform script (e.g., substituting a new curve fit algorithm) requires some knowledge of the R programming language. At the same time, transform scripts can be written in most programming languages (e.g., Perl, Python and Java), not just R.

(iv) **Certain interfaces reflect terminology specific to certain kinds of immunoassays.** The Levey-Jennings charting interface uses terminology consistent with the kind of immunoassays shown in Figure 2. For such experiments, analytes are *antigens* and the antibodies being measured are immunoglobulin *isotypes*. The interface can still be useful for other types of experiments as long as this terminology is understood.

### Future enhancements

LabKey Server undergoes ongoing development, culminating in 3–4 releases per year, so features and user interface refinements are regularly added to the platform. Features that may interest Luminex users have already been added during the period this paper has been under review. These improvements are available in LabKey Server v12.3 (released December 2012) and include: multi-curve titration plots for overlaying and comparing results from several runs (shown in Figure 10), the ability to run arbitrary numbers of transform scripts sequentially during data import, enhanced heuristics for identifying filter options for columns in data grids, and tools for anonymizing data for public release. One ongoing focus of development is support for ancillary studies [77], particularly “freezer studies” that leverage valuable, stored specimens to gain novel insights through new experiments, such as Luminex immunoassays.

## Conclusions

LabKey Server’s support for Luminex data analysis and quality control allows labs to consolidate, customize and standardize data analysis workflows, which can eliminate labor-intensive and error-prone steps. Using LabKey Server as a data repository and portal can enable teams to securely share data, analyses, quality control information, and related documents. These capabilities can be particularly helpful to research organizations whose success depends on smooth collaboration between labs, central data managers, statistical teams and principal investigators. The open source license [70] for LabKey Server and its Luminex tool allow other researchers to freely leverage, customize and improve this software.

## Availability and requirements

### LabKey Server source code and compiled binaries

The LabKey Server open source software is freely available for download at http://www.labkey.org under the terms of the Apache License 2.0 [70]. This site also provides documentation, tutorials and demos for users and developers, along with instructions for developers who wish to contribute code to the project through the LabKey Server Subversion repository.

Compiled binaries for Windows, Unix, Linux or Macintosh installation are available for free through LabKey Software at http://www.labkey.com. A graphical installer is available for computers running Windows XP or later. It includes the LabKey Server web application; the Apache Tomcat web server, v6.0.35; the Sun Java Runtime Environment, v1.7.0-29; the PostgreSQL database server, v9.2.1; and additional third-party components. Note that the installer does not include R. To access all features of the LabKey Server tool for Luminex, the R scripting environment must be installed and configured on the server.

• **Project name:** LabKey Server

• **Project home page:**http://www.labkey.org

• **Operating system(s):** Platform independent

• **Programming languages:** Java, JavaScript, R, Perl, Python, SAS, etc.

• **Other requirements, as of LabKey Server v12.3:** Apache Tomcat 6.x; Sun Java Runtime Environment 6 or 7; and either PostgreSQL (8.3, 8.4, 9.0, 9.1, or 9.2) or Microsoft SQL Server (2008 R2 or 2012 with Service Pack 1). Check the project site for latest requirements of the most recent release.

• **License:** Apache License 2.0 [70].

### Access to the Atlas Science Portal

Access to Atlas [51] is available to qualified researchers at many of the consortia participating in the Global HIV Enterprise [47,52,53]. To inquire about access, contact atlas@scharp.org.

## Abbreviations

4pl: 4 parameter logistic regression; 5pl: 5 parameter logistic regression; %CV: Coefficient of variation expressed as a percent; AIDS: Acquired immune deficiency syndrome; AUC: Area under the curve; BAMA: Binding antibody multiplex assay; EC50: Effective concentration at 50% of the difference between the asymptotes of a dose–response curve; ELISA: Enzyme-linked immunosorbent assay; HighMFI: Highest mean fluorescence intensity; HIV-1: Human immunodeficiency virus type 1; HVTN: The HIV Vaccine Trials Network; FI: Median fluorescence intensity; ID: Identifier; IgA: Immunoglobulin A; SCHARP: the Statistical Center for HIV/AIDS Research & Prevention at the Fred Hutchinson Cancer Research Center; QC: Quality control.

## Competing interests

JE, CN, EKN, EVN, and BP are employees of LabKey Software, a software consulting company that provides development, customization, and support for LabKey Server. LabKey Server is open source and freely available, so these authors do not have a direct financial interest in the software itself.

## Authors’ contributions

All authors reviewed and approved this manuscript. JE and CN designed and implemented the Luminex assay tool. JE also provided project management. EKN conceptualized, researched and wrote the paper. EKN also contributed tutorials, demos, documentation, and testing. SGS gathered requirements and provided project management and feature design. EVN contributed automated and manual testing. NLY and VCA provided usage scenarios, workflow expertise, and assistance in prioritizing laboratory requirements. LJH contributed expertise on analysis and data quality, as well as testing. MB provided data coordination and information on workflows. YF wrote the Ruminex R package. GDT saw the need for the tool and directed the lab work. BP contributed project management. JE, CN, EKN, SGS, NLY, LJH, GDT and BP contributed paper edits.

## Supplementary Material

Additional file 1 **Zip archive containing the transform script, Ruminex package, assay design archive, and supporting files.** This .zip archive can be unzipped to obtain the following files:
• LabKey transform script for Luminex: *labkey*_*luminex*_*transform*_*script*.*R*.
• Utility script used by the transform script: *youtil*.*R*.
• Ruminex R package, v0.0.9: *Ruminex*_*0*.*0*.*9*.*zip*.
• Luminex assay design archive tailored for the transform script: *Luminex Assay 200*.*xar*.
• LabKey Server list archive useful for populating fields in the assay design: *Luminex*_*ListArchive*.*lists*.*zip*.
These files have been tested against LabKey Server v12.3, released in December 2012. For future releases of LabKey Server, we recommend obtaining updated versions of these files. The most current versions are provided as part of the advanced tutorial for Luminex [55]. LabKey Server tutorials and documentation available at http://www.labkey.org are updated regularly to match the currently released version of LabKey Server. Click here for file
