# Supplementary material for: Quality control, analysis and secure sharing of Luminex® immunoassay data using the open source LabKey Server platform
Source: BMC Bioinformatics. 2013 Apr 30;14:145. doi: 10.1186/1471-2105-14-145 (PMC3671158; doi:10.1186/1471-2105-14-145)
Supplement: Additional file 1 — Zip archive containing the transform script, Ruminex package, assay design archive, and supporting files. This .zip archive can be unzipped to obtain the following files: • LabKey transform script for Luminex: labkey_luminex_transform_script.R. • Utility script used by the transform script: youtil.R. • Ruminex R package, v0.0.9: Ruminex_0.0.9.zip. • Luminex assay design archive tailored for the transform script: Luminex Assay 200.xar. • LabKey Server list archive useful for populating fields in the assay design: Luminex_ListArchive.lists.zip. These files have been tested against LabKey Server v12.3, released in December 2012. For future releases of LabKey Server, we recommend obtaining updated versions of these files. The most current versions are provided as part of the advanced tutorial for Luminex [55]. LabKey Server tutorials and documentation available at http://www.labkey.org are updated regularly to match the currently released version of LabKey Server. [file 1471-2105-14-145-S1.zip › AdditionalFile1/Ruminex_0.0.9.zip!/Ruminex/html/00Index.html]

R: R package for Luminex data analysis

# R package for Luminex data analysis

---

## Documentation for package ‘Ruminex’ version 0.0.9

- DESCRIPTION file.

## Help Pages

|  |  |
| --- | --- |
| adjust.blank.bead | Adjust non-specific binding between samples and beads using blank beads |
| fit.jags | Fit concentration-response curves with a Bayesian mixed effects model using JAGS |
| hyperparam | Hyperparameters |
| normi | Luminex Data Normalization |
| plot5PL | Plot a 5PL function |
| remove.background | Remove non-specific binding between beads and detection antibody using background well |
| rumi | Main function for the Ruminex package |
